# The effect of adsorbed lipid on pyrite oxidation under biotic conditions

**DOI:** 10.1186/1467-4866-7-8

**Published:** 2006-07-25

**Authors:** Jun Hao, Curtis Cleveland, Eelin Lim, Daniel R Strongin, Martin AA Schoonen

**Affiliations:** 1 Department of Chemistry, Temple University, Philadelphia, PA 19122, USA; 2 Department of Biology, Temple University, Philadelphia, PA 19122, USA; 3 Center for Environmental Molecular Science, Stony Brook University, Stony Brook, NY 11794-2100, USA

## Abstract

The chemolithoautotrophic bacterium, *Acidithiobacillus ferrooxidans*, commonly occurs in acid mine drainage (AMD) environments where it is responsible for catalyzing the oxidation of pyrite and concomitant development of acidic conditions. This investigation reports on the growth of this bacterial species on the pyrite surface and in the aqueous phase at a pH close to 2 as well as the role of adsorbed lipid in preventing pyrite dissolution. Both acid washed pyrite and acid-washed pyrite coated with lipids were used as substrates in the studies. The choice of lipid, 1,2-bis(10,12-tricosadiynoyl)-*sn*-Glycero-3-Phosphocholine lipid (23:2 Diyne PC), a phosphocholine lipid, was based on earlier work that showed that this lipid inhibits the abiotic oxidation rate of pyrite. Atomic force microscopy showed that under the experimental conditions used in this study, the lipid formed ~4–20 nm layers on the mineral surface. Surface-bound lipid greatly suppresses the oxidation process catalyzed by *A. ferrooxidans*. This suppression continued for the duration of the experiments (25 days maximum). Analysis of the bacterial population on the pyrite surface and in solution over the course of the experiments suggested that the pyrite oxidation was dependent in large part on the fraction of bacteria bound to the pyrite surface.

## Background

It is well-known that several species of prokaryotes are able to catalyze the oxidation of pyrite and play an important role in the development of Acid Mine Drainage (AMD), a severe environmental problem. *Acidithiobacillus ferrooxidans*, for example, utilize reduced Fe^2+ ^in AMD environments as an electron donor for energy production at low pH [1]. Due to the importance of microbes in the chemistry of such environments, a significant amount of research activity has been focused on understanding the role of microbes in the oxidation of sulfur-bearing minerals such as pyrite [2-11]. With regard to microbial-induced pyrite oxidation it is the general consensus that these microorganisms exert their impact on pyrite dissolution to a large degree by increasing the amount of available Fe^3+ ^reactant [i.e., convert Fe^2+ ^to Fe^3+^], which increases the rate of pyrite oxidation [3,12,13]. A goal of our research was to build on this prior research and to extend our understanding of microbial-accelerated pyrite oxidation to surfaces having adsorbed organic layers. With regard to this last point, the inhibition of pyrite oxidation using lipid having two hydrophobic tails (per polar head) has been studied as an AMD abatement strategy [14-16]. However, the lipid-induced inhibition of pyrite oxidation in the presence of microbes relevant to AMD has not been investigated. Toward this end, the experiments reported here were designed to investigate the effect of *A. ferrooxidans *microorganisms on pyrite oxidation in the presence and absence of adsorbed lipid layer at a solution pH near 2.

Our studies show that surface-bound lipid inhibits the oxidation of pyrite, even in the presence of bacteria. The lipid layers that suppress the oxidation limit the colonization of the bacteria on the pyrite, compared to lipid-free samples. This effect is observed throughout the duration of the experiment (about 25 days in all cases). Hence, in the presence of *A. ferrooxidans *the lipid coating remains in tact for at least 25 days and probably much longer. Furthermore, our study supports the notion that the rate of pyrite oxidation process is significantly controlled by the bacteria that colonize the pyrite surface.

## Experimental methods

Pyrite pretreatment followed protocols, described in earlier work, [15] to generate clean pyrite (~10 μm average diameter) powder with a surface area of 0.75 m^2^/g. In brief, pyrite samples were crushed and subsequently acid washed using 0.1 M HCl. Acid washing of the pyrite was carried out by flowing pH 2-deoxygenated water over the mineral powder [14].

A pure culture of *A. ferrooxidans *(ATTC 23270) was obtained from the American Type Culture Collection (Manassas, VA). The culture was grown and maintained in ATCC #2039 broth as suggested by the supplier. Briefly, the medium consisted of two separately prepared solutions: Solution A contained 0.8 g (NH_4_)_2_SO_4_, 2.0 g MgSO_4_·7H_2_O, 0.4 g K_2_HPO_4_, 5.0 mL Wolfe's Mineral Solution, and 800 mL distilled water, adjusted to pH 2.3 with H_2_SO_4 _and then filter-sterilized. Solution B contained 20.0 g FeSO_4_·7H_2_O and 200.0 ml distilled water, which was filter-sterilized and then mixed with Solution A (above) to produce the complete medium. Cultures (9 ml) for experiments were grown unshaken using 100 ml of the medium in 250 ml Erlenmeyer flasks (autoclaved at 126°C and 20 psi for 0.5 h prior to use) at 25°C. The cultures were stoppered with nylon wool wrapped with aluminum foil.

For the experiments, *A. ferrooxidans *was grown to the early stationary phase of growth and harvested by a series of filtrations. First, the bacteria were separated from particulate material, which are presumably iron hydroxide particles in the media, by filtration through a 5-μm pore size polycarbonate filter (Millipore™). Second, the bacteria in the filtrate were collected on a 0.2 μm polycarbonate filter by vacuum filtration and resuspended in sterile water [pH 2.3] by vortexing. The amount of nutrients in this cell suspension (carried over from the culture) was 1% of the original concentration. This low nutrient concentration was necessary to minimize the impact of aqueous phosphate on pyrite oxidation [17]. Nevertheless, the concentration of nutrients present was sufficient to support growth of the bacteria in the presence of pyrite.

Lipid solutions were prepared by techniques described elsewhere [14]. In the current study, 0.1 g of acid washed pyrite was added to lipid solution (20 ml). The lipid solution contained 7.5 μmol of 23:2 Diyne PC lipid (Avanti Polar Lipids), which has the following structure:



This particular concentration of lipid was chosen on the basis of prior research in our laboratory, and it was sufficient to cover the majority of the pyrite surface [14]. After 1 hour of mixing, the pyrite slurry was allowed to settle and the supernatant, which contained free lipid, was subsequently removed by decanting. The remaining fraction of lipid-coated pyrite slurry was used in the experiments.

The experiment was performed on two separate dates (from here on referred to as Experiment 1 and 2). The experimental conditions, treatments and analyses for both experiments were identical with the exception of the starting bacterial densities (see below). The treatments consisted of: 1) pyrite, 2) lipid-coated pyrite, 3) pyrite + bacteria, 4) lipid-coated pyrite + bacteria. All the treatments were prepared, in duplicate, in distilled water (pH 2.0) for a total volume of 50 ml. The treatments with pyrite contained 0.1 g of pyrite. Approximately the same amount of pyrite was present in the pyrite/lipid treatments (see preparation of lipid-coated pyrite slurry described above). The final density of *A. ferrooxidans *in the treatments with bacteria was approximately 3.3 × 10^7^cells/ml for Experiment 1 and 1.7 × 10^7^cells/ml for Experiment 2. Each treatment mixture was stirred continuously (using a teflon coated stir bar) throughout the experiment, which lasted a maximum of 25 days. Aliquots (1 ml) were withdrawn every 2–3 days for cell count analysis and preserved in 3.7% formaldehyde. In addition, 0.1 ml aliquots were withdrawn to determine the iron concentration in solution, which was used as a reaction progress variable. Total iron solution concentration determinations (including ferrous and ferric iron) were determined spectrophotometrically by using the ferrozine technique (UV method 8008). Since this technique is intrinsically sensitive to the concentration of ferrous, ascorbic acid was added to the solution prior to analysis to reduce any ferric to ferrous. All the sample aliquots for the ferrozine test were filtered through a 0.2 μm polycarbonate filter before analysis. Solution pH measurements were carried out with a PHM82 standard meter with a resolution of 0.01 pH units.

Enumeration of bacterial cell densities in solution and on pyrite powder were performed by epifluorescence microscopy following staining with the fluorochrome dye, acridine orange (AO) [18,19]. Briefly, preserved water samples were vacuum filtered onto 0.2-μm-pore-size blackened polycarbonate filters (Nuclepore) and the cells collected on the filter were stained with AO at a final concentration of 0.01% for 3 min. The volume of sample filtered for cell counts was chosen such that the layer of pyrite particles that accumulated on the filter would not obscure the bacteria. Cells were observed and counted with a Zeiss standard microscope equipped for epifluorescence microscopy. Filter sets used for AO fluorescence observations are as follows: a BP450-490 exciter filter, and FT510 chromatic beam splitter, and an LP520 barrier filter. For each sample, the number of bacteria on 10 to 15 randomly chosen fields distributed over the filter (or approximately 300 to 600 cells in total per filter) was counted. To determine the number of cells bound to pyrite, 0.1 ml aliquots (initial total volume was 50 ml) of the bacteria/pyrite solutions were collected on a 5 μm polycarbonate membrane filter (Millipore). The particles were subsequently washed so that any bacteria in solution would pass through the filter. The density of cells on the particulate material remaining on the filter was then determined using the staining method described above.

Surfaces for AFM were prepared from pyrite cubes (Navajun, Spain) obtained from Ward's Natural Science. Growth surfaces were cut from the cube and were subsequently polished. Prior to an experiment, the samples were exposed to pH 2 water (by HCl addition) to remove oxidized layers prior to exposing to lipid solution. To prepare the lipid/pyrite samples the pyrite platelet was submerged into the lipid solution in a vertical orientation (via a leading edge). The sample was then immediately removed, washed with pH 2 water to remove any loosely bound lipid, and then reintroduced to pH 2 water for imaging with a PicoSPM II (Molecular Imaging) microscope. The probes used in all the AFM measurements [NSC15, μMasch] had a nominal spring constant of 40 N/m and a resonant frequency of 325 kHz.

## Results and discussion

It is generally accepted that both dissolved molecular oxygen and aqueous Fe^3+ ^play a role in the oxidation of pyrite [12,20]. The overall composite reactions for pyrite oxidation in the presence of both these oxidizing agents can be expressed as follows:

FeS_2 _+ (7/2)O_2 _+ H_2_O → Fe^2+ ^+ 2SO_4 _^2- ^+ 2H^+ ^    (1)

FeS_2 _+ 14Fe^3+ ^+ 8H_2_O → 15Fe^2+ ^+ 2 SO_4 _^2- ^+ 16H^+ ^    (2)

Prior studies of pyrite oxidation have shown that Fe(III) is a more aggressive oxidant of the pyrite surface than dissolved molecular oxygen [21,22]. Under strictly abiotic conditions, however, the concentration of Fe^3+ ^in solution is relatively low, due to the slow oxidation kinetics associated with the conversion of Fe^2+ ^product [see Eqn. (1)] to Fe^3+ ^by dissolved O_2_. Hence, the contribution of eqn. (2) to pyrite oxidation under abiotic conditions is limited by the rate of ferrous iron oxidation, which is low in pH 2 solutions [21]. It is precisely this slow abiotic oxidation step that is catalyzed by microbes, such as *A. ferrooxidans*. The subsequent oxidation of the mineral by the aqueous Fe^3+ ^is referred to as the "indirect" mechanism of pyrite oxidation [20]. This mechanism is in contrast to the "direct" mechanism, that involves the oxidation of the pyrite by surface colonized bacteria (either by enzymatic or non-enzymatic means) [8,20].

**Figure 1:**
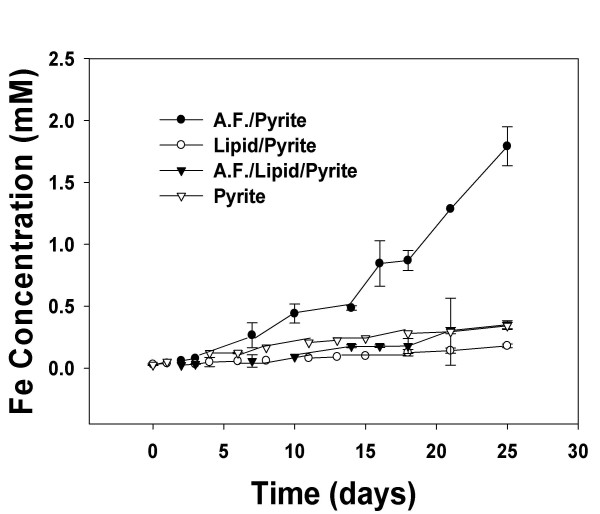
The amount of oxidation, based on the solution concentration of Fe, for the various pyrite systems. The initial cell density for the bacteria was 3.3 × 10^7^cells/ml. All the systems were initially at a pH of 2.0. A.F. denotes *A. ferrooxidans*.

Data plotted in Figure 1 exhibits the influence of bacteria on the rate of pyrite oxidation. Included in this figure are experiments with and without lipid. In all these experiments, total dissolved iron is used as the progress variable, which is appropriate for these conditions because the concentration of both ferrous and ferric iron is not limited by the precipitation of an iron oxide at the low solution pH maintained in these experiments [23].

There are at least three important observations that can be made. First, the rate of oxidation is the highest for experiments in which pyrite without a lipid pretreatment is exposed to *A. ferrooxidans*. Second, if the pyrite is exposed to lipid, prior to the exposure to the bacteria, there is a significant decrease in the amount of pyrite oxidation. Specifically, over the last 10-day period (where the solution is supporting the steady growth of the bacteria; see below) of the experiment, there is a >4-fold decrease in the oxidation rate when pyrite has an adsorbed lipid layer. Third, while lipid suppresses the amount of bacterial-induced pyrite oxidation, the rate of oxidation in the presence of microbes is higher than in the absence of microbes. Hence, the presence of the lipid on the pyrite surface does not entirely suppress the influence of the bacteria. For example, over the last 10-day period, the amount of oxidation associated with the bacteria/lipid/pyrite system was a factor of three higher than the rate of oxidation associated with the abiotic lipid/pyrite system, and it was very similar (within experimental error) to the rate of oxidation associated with untreated pyrite in pH 2 water (i.e., no bacteria or lipid).

A summary of these data, including the amount of suppression exhibited by the lipid coating over the last 10 days of each experimental run in the biotic and abiotic environments are given in Table 1. The rates measured in this study of 2.1 × 10^-9 ^and 1.62 × 10^-8 ^mol/m^2^-s at pH 2 for the abiotic and biotic circumstance, respectively, are in reasonable agreement with prior studies that investigated the oxidation of pyrite in the presence of *A. ferrooxidans*. Differences in pH, cell density, mineral surface area, and growth media between studies all lead to variability in the measured abiotic and biotic rates. Prior research by Olson, for example, measured abiotic and biotic oxidation rates of ~2 × 10^-9 ^and ~9 × 10^-8 ^mol/m^2^-s, respectively at a pH close to 2, [24] while a recent study at a similar pH measured the biotic rate to be 7 × 10^-10 ^mol/m^2^-s [13]. We emphasize that while our measured rate fall within the spread associated with prior studies, perhaps more important to this study is that the rate of pyrite oxidation in the presence of bacteria can be significantly suppressed when phosphochloine lipid is adsorbed on pyrite (a rate decrease from 1.62 × 10^-8 ^to 3.1 × 10^-9 ^mol/m^2^-s).

**Table 1:** Summary of experimental observations for the amount of pyrite oxidation in the presence of lipid and bacteria.

**Samples**	**Lipid Amount**	Oxidation rate* (10^-8^M s^-1^m^-2^)	**% Oxidation Suppression**^#^
*A. ferrooxidans*/Pyrite	N/A	1.62 ± 0.1	N/A
*A. ferrooxidans*/Lipid/Pyrite	7.5 μmol	0.30 ± 0.02	81
Lipid/Pyrite	7.5 μmol	0.088 ± 0.005	95
Pyrite	N/A	0.21 ± 0.01	N/A

N/A denotes Not Applicable

*Rates are estimated from a linear regression analysis of the final 10 days of data in Figure 1.

^#^Values are relative to *A. ferrooxidans*/Pyrite rate.

**Figure 2:**
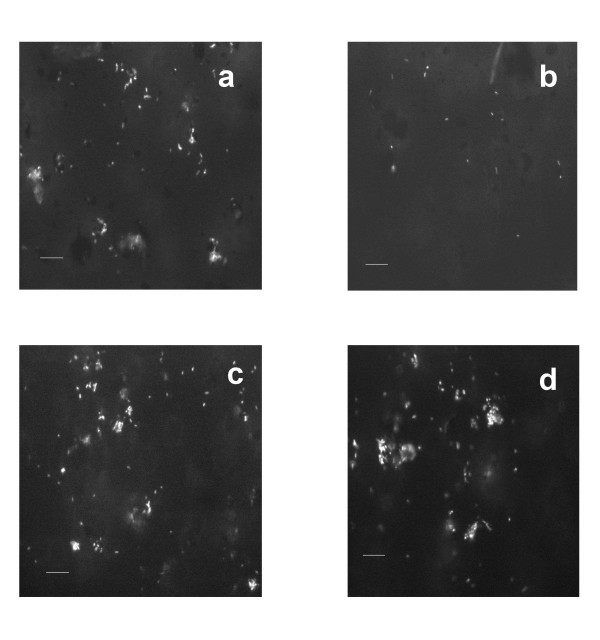
Epifluorescent images (microbes appear as bright spots) of pyrite powder after various reaction conditions. (a) Pyrite exposed to bacteria for 10 days; (b) lipid/pyrite exposed to bacteria for 10 days; (c) a different pyrite sample exposed to bacteria for 10 days and the (d) same sample after vortexing. These images suggest that *A. ferrooxidans *colonize untreated pyrite surfaces and that the presence of lipids inhibits this colonization. The scale bar for all the images is 10 μm.

In order to understand the role of bacteria in promoting pyrite oxidation in systems treated with lipids it is useful to analyze the distribution of bacteria between the mineral and the solution phase. As a first step toward this analysis it is necessary to confirm that cells measured by microscopy as being bound to the surface are in fact truly mineral-bound and not a superficial bacterial layer that results from the preparation of the samples. Epifluorescence images of the bacterial/pyrite samples illustrate these differences in bacterial attachment (Figure 2). Images 2a and 2b compare the bacteria/pyrite and bacteria/lipid/pyrite samples, respectively, after the samples were exposed to oxidizing conditions in solution for 10 days. The pyrite particles are not visible with epifluorescence microscopy (because the fluorochrome dyes used for staining cells specifically bind to protein and/or DNA only), and bacteria that are attached to the pyrite are visible as microcolonies (cell aggregates). This observation is common when visualizing surface-bound cells on organic or inorganic particles by epifluorescence microscopy. Such microcolonies of surface-bound bacteria were abundant in the pyrite/bacteria sample (Image 2a) but not the pyrite/lipid/bacteria sample (Image 2b) (quantification of images to determine cell densities was also performed and is presented below.) These images suggest that the presence of the lipid impedes the interaction of the bacteria with the pyrite surface. To support our contention that the bacteria are actually bound to the pyrite particles, the samples were vigorously vortexed prior to imaging (see Figure 2c and 2d). After this treatment, the bacterial aggregates were still present, suggesting a strong adhesion of the microbes to the pyrite surface. We argue that if a bacterium simply settled on the pyrite surface during preparation (see methods) it would have been removed by the additional vortexing and washing.

**Figure 3:**
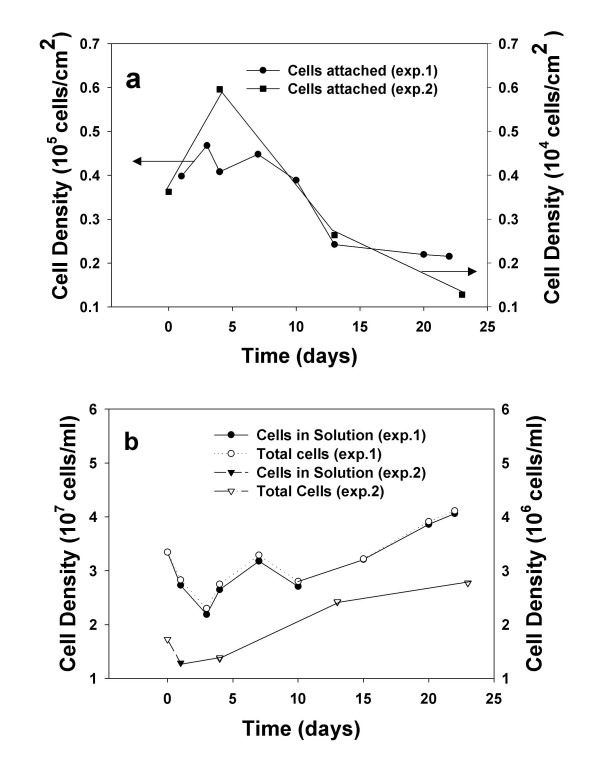
Cell density plots for (a) bacteria bound to lipid/pyrite, (b) bacteria in solution for lipid/pyrite as a function of time. Data is plotted for two independent experiments, denoted by exp. 1 (left y-axis) and exp. 2 (right y-axis).

**Figure 4:**
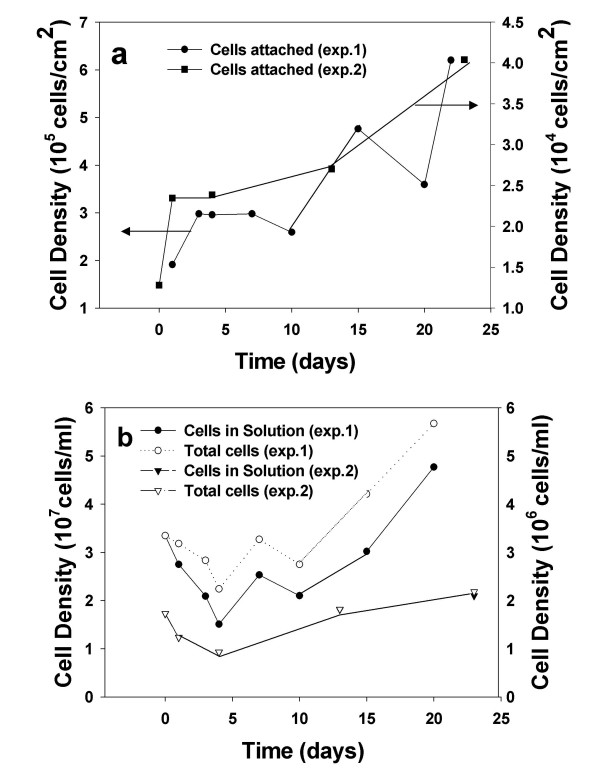
Cell density plots for (a) bacteria bound to pyrite, and (b) bacteria in the contacting solution as a function of time. Data is plotted for two independent experiments, denoted by exp. 1 (left y-axis) and exp. 2 (right y-axis).

It is also important to address the issue of the viability of the bacteria under our experimental conditions. Our staining technique presumably counts both living and dead cells, and hence, our cell densities should be taken as an upper limit. While we cannot distinguish the viable from non-viable fraction of bacteria, we can assert that a significant fraction of the bacteria fall in the former category, based on at least two experimental observations; 1) the rate of pyrite oxidation is greatly enhanced by the presence of bacteria (Table 1 and Figure 1) and 2) the surface and solution cell community populations increase with time for the lipid-free circumstance (Figure 3 and 4, below).

Figure 3 shows the distribution of *A. ferrooxidans *density between the surface bound and solution fraction as a function of time for the bacteria/lipid/pyrite system. Independent data sets for the two experimental runs are shown, and aside from differences in the initial cell densities at time-zero, the data from both experiments reveal similar trends. After a slight decrease in bacterial density in the bacteria/lipid/pyrite treatment over the initial 5 days (most evident in Figure 3b), there is an increase in the solution concentration of bacteria and a decrease in the surface-bound fraction (Figure 3a and 3b). We attribute the initial decrease of bacteria in solution to the lack of substrate (the treatments were prepared in DI water) required to support bacterial growth during the early stages of the experiment. Presumably, there is residual uncoated (i.e., lipid-free) pyrite present and its dissolution at the beginning of the experiment starts to provide nutrients for the bacteria so that a net growth can be achieved after the 5-day period. This experimental observation also is the reason why Table 1 compiles oxidation rate data for the last 10-day period of each experiment. In contrast, the surface bound fraction of bacteria shows an experimentally resolvable decrease between 5 and 20 days when lipid is present.

Figure 4a and 4b exhibit bacterial density measurements for the bacteria/pyrite system (no lipid). Again two independent experimental data sets are shown. While the solution density of bacteria in the pyrite and lipid/pyrite system is similar [compare Figure 4b (Exp. 1) to Figure 3b (Exp. 1)], the bacterial density on the surface of the lipid-free pyrite after 20 days is more than a factor of 10 greater than that of the lipid/pyrite system [compare Figure 4a (Exp. 1) to Figure 3a (Exp. 1)]. Additionally, the surface-bound fraction in the lipid-free circumstance continues to grow over the entire 25-day experiment, in contrast to the decrease associated with the lipid-coated pyrite case. Perhaps, the most revealing experimental observations concerned with the data presented in Figure 3 and 4 are as follows (N.B., values associated with Exp. 1 are used in the following discussion for convenience). First, the solution concentration of bacteria in the bacteria/pyrite system after the 20-day period is approximately 15% higher than in the bacteria/lipid/pyrite system (4.8 × 10^7^compared to 4.1 × 10^7 ^cells/ml, respectively). Second, after this same time period, the attached cell density for the bacteria/pyrite system is more than an order of magnitude (20×) higher than for the bacteria/lipid/pyrite system (6.4 × 10^5 ^compared to 2.3 × 10^4 ^cells/cm^2^, respectively). Furthermore, the attached cell density for the lipid pyrite system actually decreases over the 20-day period (from 4.0 × 10^4 ^to 2.3 × 10^4 ^cells/cm^2^), while the surface cell density for the lipid-free system underwent more than a three-fold increase (from 1.9 × 10^5 ^to 6.4 × 10^5 ^cells/ml). These data should be considered in view of the rate data compiled in Table 1, which shows that the oxidation rate for the bacteria/pyrite system is more than a factor of 4 greater than the bacteria/lipid/pyrite system. Since, the solution concentration of bacteria is within about 15% for both these systems, it is difficult to attribute the difference in oxidation rate entirely to differences in the concentration of this fraction of bacteria. Instead, we argue that the difference in oxidation rate between the bacteria/lipid/pyrite and bacteria/pyrite systems must be largely due to differences in the concentrations of surface colonized bacteria for the two systems. Only the lipid-free pyrite shows a significant increase in the surface-bound fraction of bacteria, in contrast to the decreasing concentration of surface-bound bacteria on lipid/pyrite over the course of the experiment. Prior studies have in general shown the indirect mechanism to dominate, [3,13] but during the early stages of the pyrite oxidation process the relative contribution of the direct mechanism can be significant [25]. We suspect that the growth rates of the surface attached and solution phase bacteria achieved in our study (which are non-exponential growth rates) are consistent with an early stage of the oxidation process, where bacterial growth in solution is limited by the availability of aqueous Fe^2+ ^(resulting from the dissolution of the mineral surface). In general, the importance of the surface-bound bacterial fraction for pyrite oxidation is well appreciated by prior research. It has already been shown in prior research, for example, that bacteria attached to the pyrite surface has significant effects on the oxidation process, due in part to the microbe's influence on the evolution of the mineral surface and contacting solution [7,26,27].

**Figure 5:**
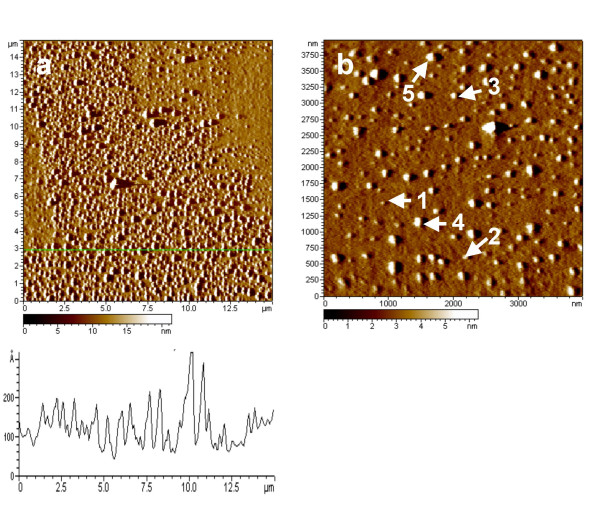
AFM images of lipid covered pyrite in two different regions of surface. (a) A 15 × 15 micron scan showing a region with a heterogeneous, but relatively high lipid coverage. (b) A 4 × 4 micron scan of a region of the pyrite that had a lower lipid coverage. Certain features are labeled to emphasize the variation of lipid island heights. Features 1, 2, 3, 4, and 5 have heights of 4, 7, 10, 15, and 20 nm, respectively.

In an effort to better characterize the structure of the lipid layer on pyrite, we carried out AFM experiments in the absence of bacteria that investigated the adsorption of the lipid on pyrite platelets. In contrast to the pyrite powder, the comparatively flat platelets are more conducive to AFM imaging. Figure 5 exhibits two images of pyrite that had been exposed to the 23:2 dyne lipid at solution concentrations of lipid that were similar to those conditions used for our pyrite powder studies. Figure 5a is a 15 × 15 μm scan and shows a rather "patchy" lipid coverage on the surface, suggesting that at the conditions used in our experiments, some of pyrite surface is left lipid-free. An analysis of the topography of the features present in the image shows that the highest features extend ~20 nm above the baseline in the image, but the majority of the lipid features exhibit height values in the range of 7–16 nm (features as small as 4 nm are present). The baseline height in this image, however, may be an underlying lipid layer or the pyrite surface. To address this height issue, we present the image in Figure 4b that is associated with a different part of the pyrite surface, that has a lower coverage of lipid, and allows the bare pyrite surface to be identified with more certainty. The height of the lipid features associated with this image range from about 4–20 nm, similar to the heights of the features associated with the more concentrated lipid layer exhibited in Figure 5a.

The mechanism by which the lipid suppresses the pyrite oxidation reaction appears to be connected to the formation of a surface coating with a hydrophobic pocket. The AFM analysis of this coating shows a relatively thin coating ranging from 7 to 16 nm, within the experimental error of our measurement. Prior results in our laboratory suggested that another phosphocholine lipid, L-α-Phosphatidylcholine, Hydrogenated (Egg, Chicken), assembles into a bilayer structure on the pyrite surface. Such a structure would consist of a phosphate group in a phosphocholine headgroup binding to the mineral, and a second phosphate group extending into the aqueous phase, leaving an hydrophobic interior. This prior model was first deduced from vibrational studies of the lipid/mineral interaction, [15] but more recent atomic force microscopy (AFM) results lend support to this model [28]. In particular, imaging of the lipid/pyrite (platelet sample) surface at similar conditions to those used in the prior study (i.e., pyrite surface area to lipid concentration ratio) find that the layer height of the lipid is consistent with what would be expected for a bilayer, although these structures occupy the surface along with thicker or mulitilayer structures [28]. Our imaging results of the 23:2 Diyne lipid in the present study are consistent the presence of bilayer structures. The chain length of the particular lipid used in this study is approximately 3.5 nm. Hence, a bilayer might of been expected to be about 7 nm, or two stacked bilayers would be ~14 nm, similar to some of our experimentally determined heights. Certainly, a more detailed study of the surface is needed to make any conclusive arguments concerning the existence of a bilayer structure. Perhaps, more importantly, we infer from our AFM results that the pyrite powder used in our study would likewise be covered by a lipid coating under similar conditions. We further argue that this coating both limits the amount of microbial adhesion and the reaction of oxidants, such as Fe^3+^, and water with the mineral surface. Finally, the results from the present study also show that the 23:2 Diyne PC structure is stable in the presence of *A. ferrooxidans *under our experimental conditions for at least a period of 25 days.

## Summary

The amount of pyrite oxidation in the presence of bacteria and with an adsorbed phosphocholine layer has been investigated. The presence of *A. ferrooxidans *greatly increases the amount of pyrite oxidation relative to the bacteria-free system. Pretreatment of pyrite with the phosphocholine lipid reduces its oxidation rate compared to untreated pyrite. By measuring the density of surface bound and solution bacteria it was determined that under our experimental conditions the amount of pyrite oxidation is more a function of the surface-colonized bacteria concentration than the solution bacterial fraction. Our results also show that the lipid oxidation barrier layers are stable in the presence of *A. ferrooxidans *for at least 25 days under our experimental conditions. A separate study will be conducted to evaluate the fate of the surface-bound lipids in the presence of heterotrophic bacteria.

## References

[B1] Santelli CM, Welch SA, Westrich HR, Banfield JF (2001). The effect of Fe-oxidizing bacteria on Fe-silicate mineral dissolution. Chemical Geology.

[B2] Chakrabarti BK, Banerjee PC (1991). Surface hydrophobicity of acidophilic heterotrophic bacterial cells in relation to their adhesion on minerals. Canadian Journal of Microbiology.

[B3] Southam G, Beveridge TJ (1992). Enumeration of thiobacilli within pH-neutral and acidic mine tailings and their role in the development of secondary mineral soil. Applied and Environmental Microbiology.

[B4] Dziurla M-A, Achouak W, Lam B-T, Heulin T, Berthelin J (1998). Enzyme-linked immunofiltration assay to estimate attachment of thiobacilli to pyrite. Applied and Environmental Microbiology.

[B5] Ohmura N, Kitamura K, Saiki H (1993). Selective adhesion of Thiobacillus ferrooxidans to pyrite. Applied and Environmental Microbiology.

[B6] Lawrence JR, Kwong YTJ, Swerhone GDW (1997). Colonization and weathering of natural sulfide mineral assemblages by Thiobacillus ferrooxidans. Canadian Journal of Microbiology.

[B7] Edwards KJ, Schrenk MO, Hamers R, Banfield JF (1998). Microbial oxidation of pyrite: experiments using microorganisms from an extreme acidic environment. American Mineralogist.

[B8] Sand W, Gerke T, Hallmann R, Schippers A (1995). Sulfur chemistry, biofilm, and the (in)direct attack mechanism – a critical evaluation of bacterial leaching. Applied Microbiology and Biotechnology.

[B9] Wakao N, Mishina M, Sakurai Y, Shiota H (1984). Bacterial pyrite oxidation. III. Adsorption of Thiobacillus ferrooxidans cells on solid surfaces and its effect on iron release from pyrite. Journal of General and Applied Microbiology.

[B10] Edwards KJ, Bond PL, Banfield JF (2000). Characteristics of attachment and growth of Thiobacillus caldus on sulfide minerals: a chemotactic response to sulfur minerals?. Environmental Microbiology.

[B11] Mielke RE, Pace DL, Porter T, Southam G (2003). A critical stage in the formation of acid mine drainage: colonization of pyrite by Acidithiobacillus ferrooxidans under pH-neutral conditions. Geobiology.

[B12] Nordstrom DK, Southam G (1997). Geomicrobiology of sulfide mineral oxidation. Reviews in Mineralogy.

[B13] Gleisner M, Herbert RB, Frogner Kockum PC (2006). Pyrite oxidation by Acidithiobacillus ferrooxidans at various concentrations of dissolved oxygen. Chemical Geology.

[B14] Elsetinow AR, Borda MJ, Schoonen MAA, Strongin DR (2003). Suppression of pyrite oxidation in acidic aqueous environments using lipids having two hydrophobic tails. Advances in Environmental Research.

[B15] Zhang X, Borda MJ, Schoonen MAA, Strongin DR (2003). Adsorption of phospholipids on pyrite and their effect on surface oxidation. Langmuir.

[B16] Zhang X, Borda MJ, Schoonen MAA, Strongin DR (2003). Pyrite oxidation inhibition by a cross-linked lipid coating. Geochemical Transactions.

[B17] Elsetinow AR, Schoonen MAA, Strongin DR (2001). Aqueous geochemical and surface science investigation of the effect of phosphate on pyrite oxidation. Environmental Science and Technology.

[B18] Muyer G, de Bruyn JC, Schmedding DJM, Bos P, Westbroek P, Kuenen JG (1987). A combination immunofluorescence-DNA-fluorescence staining technique for enumeration of Thiobacillus ferrooxidans in a population of acidophilic bacteria. App Environ Microbiol.

[B19] Sherr BF, Sherr EB, del Giorgio P, Paul J (2001). Enumeration of total and highly active bacteria. Marine Microbiology.

[B20] Silverman MP (1967). Mechanism of bacterial pyrite oxidation. Journal of Bacteriology.

[B21] Singer PC, Stumm W (1970). Acid mine drainage: the rate-limiting step. Science.

[B22] Moses CO, Herman JS (1991). Pyrite oxidation at circumneutral pH. Geochimica et Cosmochimica Acta.

[B23] Schoonen M, Elsetinow A, Borda M, Strongin D (2000). Effect of temperature and illumination on pyrite oxidation between pH 2 and 6. Geochem Trans.

[B24] Olson GJ (1991). Rate of pyrite bioleaching by Thiobacillus ferrooxidans: results of an interlaboratory comparison. Applied and Environmental Microbiology.

[B25] Yu JY, McGenity TJ, Coleman ML (2001). Solution chemistry during the lag phase and exponential phase of pyrite oxidation by Thiobacillus ferrooxidans. Chemical Geology.

[B26] Edwards KJ, Goebel BM, Rodgers TM, Schrenk MO, Gihring TM, Cardona MM, Hu B, McGuire MM, Hamers RJ, Pace NR, Banfield JF (1999). Geomicrobiology of pyrite (FeS2) dissolution: case study at Iron Mountain, California. Geomicrobiology Journal.

[B27] Pace DL, Mielke RE, Southam G, Porter TL (2005). Scanning Force Microscopy Studies of the Colonization and Growth of A. ferrooxidans on the Surface of Pyrite Minerals. Scanning.

[B28] Zhang XV, Kendall TA, Hao J, Strongin DR, Schoonen MAA, Martin ST (2006). Physical structures of lipid layers on pyrite. Environmental Science and Technology.

